# eGFR and deep white matter hyperintensity as predictors of cognitive decline long after carotid endarterectomy

**DOI:** 10.1038/s41598-019-54459-6

**Published:** 2019-11-28

**Authors:** Akira Nakamizo, Toshiyuki Amano, Takahiro Kuwashiro, Masahiro Yasaka, Yasushi Okada

**Affiliations:** 1 Department of Neurosurgery, Clinical Research Institute, National Hospital Organization Kyushu Medical Center, Fukuoka, Japan; 2 Department of Cerebrovascular Medicine and Neurology, Clinical Research Institute, National Hospital Organization Kyushu Medical Center, Fukuoka, Japan

**Keywords:** Cerebrovascular disorders, Risk factors, Cognitive neuroscience

## Abstract

Chronic kidney disease and white matter hyperintensity (WMH) are associated with cognitive decline. The aim of this study was to assess the correlations between estimated glomerular filtration rate (eGFR) or WMH and cognitive function in patients who have undergone carotid endarterectomy (CEA). Cognitive functions were investigated using the Neurobehavioral Cognitive Status Examination (Cognistat) in 83 patients who had undergone CEA. The eGFR at 5 years prior to examination was significantly associated with severe cognitive impairment (odds ratio, 0.89 per 1-mL/min/1.73 m^2^ increase, 95% confidence interval 0.82–0.97, p = 0.0004). Receiver operating characteristic analysis revealed that a cutoff eGFR of 46.8 mL/min/1.73 m^2^ at 5 years prior to examination offered the most reliable predictor of severe cognitive impairment (sensitivity 88.9%, specificity 76.5%, area under the curve 0.848). The eGFR at 5 years prior to examination showed a significant linear association with total Cognistat score (r^2^ = 0.11035, p = 0.0032) compared to eGFR at 3 years prior to examination (r^2^ = 0.06455, p = 0.0230) or at examination (r^2^ = 0.0210, p = 0.0210). Spearman’s correlation coefficient revealed that orientation, comprehension, repetition, construction, memory, and similarity correlated with eGFR at 5 years prior to examination. Conversely, Fazekas grade for deep WMH at examination was associated with total Cognistat score (p = 0.0016), unlike that at 3 years (p = 0.0100) or 5 years prior to examination (p = 0.0172). While eGFR correlates with future cognitive function, deep WMH associates with present cognitive function in patients who have undergone CEA.

## Introduction

Carotid stenosis shares various risk factors common for cognitive decline such as hypertension, diabetes, and dyslipidemia, but cognitive function is rarely considered in evaluating or treating carotid stenosis. Carotid endarterectomy (CEA) might theoretically improve or maintain cognitive function by restoring normal cerebral perfusions and by removing the source of microemboli immediately after surgery. However, CEA reduced microemboli only up to 70% at 1 year after surgery in 28 patients^1^, suggesting that risk of cognitive decline persists even after CEA. Moreover, microemboli during surgical maneuvers, hypoperfusion during carotid artery clamping, general anesthesia, and postoperative hyperperfusion can cause postoperative cognitive declines^2^. Taken together, CEA can result in improvement or deterioration of cognitive function, and whether these complex interactions ultimately result in cognitive improvement or decline remains unclear^3^. In the long term, various cerebrovascular risk factors might influence cognitive function after CEA. Periodic cognitive assessment thus appears necessary to detect early cognitive changes long after CEA.

Several studies have reported that age, education, intelligence quotient, brain atrophy, preoperative symptoms, and white matter hyperintensity (WMH) are associated with cognitive changes after CEA, but most such studies were performed in the early postoperative stage, mainly at only 1–6 months after CEA^4–6^. CEA prevented expected increases in WMH at 1 year after CEA in 14 patients with preoperative cognitive decline^1^, but the effects of the existing WMH on long-term cognitive change are uncertain. Chronic kidney disease (CKD) is also known to increase the risk of cognitive decline^7,8^. Low estimated glomerular filtration rate (eGFR) was associated with dysexecutive and amnestic mild cognitive impairment in 622 subjects who were ≥70 years old^9^, but little is known about the association between eGFR and cognitive function long after CEA.

The aim of this study was to assess the associations between eGFR or WMH and cognitive function in patients who have undergone CEA, particularly in terms of whether eGFR and WMH are useful to predict cognitive declines occurring within several years.

## Material and Methods

### Patients

**Figure 1:**
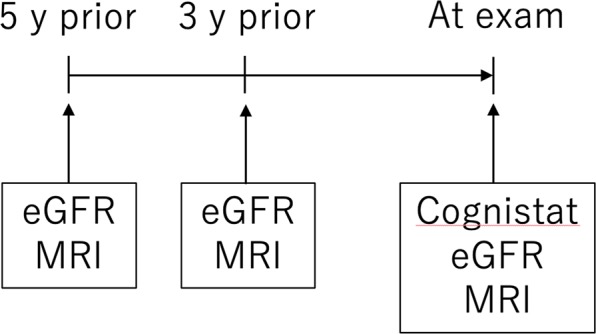
Flowchart of the study.

Eighty-three patients who had undergone CEA and were admitted to our institution from February 2016 to October 2018 for periodic follow-up were invited to participate in this study. This study was approved by the National Hospital Organization Kyushu Medical Center Ethical Review Board and performed in accordance with the relevant guidelines and regulations, and all patients provided written informed consent prior to enrolment. This study represented a retrograde analysis of prospectively collected data. Figure 1 shows a flowchart for participation in this study. All patients underwent CEA for severe carotid stenosis or unstable plaque under general anesthesia with propofol. Surgical indications for carotid stenosis were as follows: linear stenosis >70%, area stenosis >90%, and peak systolic flow velocity of cervical internal carotid artery (ICA) > 200 cm/s on color-coded sonography. Twenty-three patients with carotid stenosis >50% who were treated conservatively were also investigated as a reference group.

### Cognitive assessment

The Japanese versions of Cognistat^10^ was adopted to assess cognitive functions. Cognistat consists of 10 sub-components: orientation, attention, comprehension, repetition, naming, construction, memory, calculation, similarities, and judgment^11^. Severity of impairment in each cognitive domain is defined as follows: score ≥9, normal; 8, mild impairment; 7, moderate impairment; and ≤6, severe impairment^10^. Patients who had undergone Cognistat previously were not included in this study, to avoid any practice effect.

### Magnetic resonance imaging (MRI)

All patients underwent 1.5-T MRI to assess brain WMH. Deep WMH and periventricular hyperintensity (PVH) on T2-weighted imaging were classified using Fazekas grade^12^. Intracranial atherosclerosis was defined as >50% stenosis of the ICA, middle cerebral artery (MCA), or anterior cerebral artery (ACA) on magnetic resonance angiography (MRA).

### Ultrasound

All patients underwent cervical ultrasound to measure carotid stenosis using the European Carotid Surgery Trial method. Postoperative hyperperfusion state was defined as a >1.5-fold increase in mean flow velocity of the MCA compared to the preoperative value on transcranial color-coded sonography.

### Statistical analysis

Except where indicated otherwise, all results are reported as mean ± standard deviation. Statistical analysis was performed using the Wilcoxon rank-sum test for non-parametric data. Differences in binary variables were assessed using Pearson’s chi-square test. Logistic regression models were used to evaluate independent associations between variables and cognitive decline. Cutoff values were defined for eGFR. Sensitivity and specificity were calculated to predict severe impairment on Cognistat. Linear regression analysis was performed for paired eGFR and Cognistat scores. The correlation between Cognistat scores and eGFR was assessed using Spearman’s correlation coefficient. Kruskal-Wallis test was used to assess differences in Cognistat scores between Fazekas grades for deep WMH. Differences were considered significant for values of p < 0.05. All statistical analyses were performed using JMP Pro version 13.2 software (SAS Institute, Cary, NC).

## Results

### Patients

Participants comprised 83 patients with a history of CEA (68 men, 15 women; mean age at examination, 76.5 ± 5.9 years; mean follow-up between CEA and examination, 6.7 ± 3.1 years). Preoperative presentation was symptomatic in 50 patients (60.2%), comprising brain infarction in 30, transient ischemic attack in 11, and monocular blindness in 8, and seizure in 1. Among symptomatic patients, mean interval from onset to CEA was 2.2 months. No significant differences in the number of severely impaired cognitive domains on Cognistat or total Cognistat score were seen between symptomatic and asymptomatic patients (p = 0.5489 and p = 0.6647, respectively). During follow-up, two patients suffered ipsilateral lacunar infarction, at 5 and 7 years after CEA, but no patients experienced recurrent ipsilateral symptoms due to restenosis. Eighteen patients (21.7%) showed brain infarction >3 cm in maximum diameter at cognitive examination. No significant differences in the number of severely impaired cognitive domains on Cognistat or total Cognistat score were seen between patients with and without brain infarction >3 cm in maximum diameter (p = 0.6937 or p = 0.2385, respectively).

### Risk factors and cognitive impairment

Table 1 shows baseline demographic characteristics by the number of severely impaired domains on Cognistat. At least one domain was severely impaired in 47 patients, ≥2 domains in 19 patients, and ≥3 domains in 10 patients. The eGFR was significantly low and deep WMH grade was significantly high in patients with severe impairment in ≥3 domains compared to other patients. This difference was significant for eGFR at 5 years prior to examination compared to eGFR at 3 years prior to or at examination. Conversely, the difference between deep WMH grade at examination and deep WMH grade at 5 years or 3 years prior to examination was significant.

**Table 1:** Baseline demographic characteristics.

	Overall (n = 83)	Number of severely impaired cognitive domains on Cognistat				
		None (n = 36)	1 (n = 28)	2 (n = 9)	≥3 (n = 10)	P value
Age at examination (years)	76.5 ± 5.9	75.9 ± 5.8	74.1 ± 5.5	81.1 ± 3.9	81.2 ± 3.5	0.0002
Education (years)	12.9 ± 2.9	14.2 ± 2.1	12.5 ± 2.8	10.8 ± 3.4	11.6 ± 3.3	0.0056
Follow-up period (years)	6.7 ± 3.1	6.3 ± 2.8	6.8 ± 3.2	6.4 ± 3.0	8.2 ± 3.9	0.5533
Male (%)	68 (81.9)	29 (80.6)	22 (78.6)	9 (100)	8 (80.0)	0.5184
Symptomatic presentation (%)	50 (60.2)	20 (55.6)	18 (64.3)	7 (77.8)	5 (50.0)	0.5489
**Medical history (%)**						
Hypertension	78 (94.0)	36 (100)	25 (89.3)	7 (77.8)	10 (100)	0.0419
Diabetes mellitus	40 (48.2)	17 (47.2)	14 (50.0)	5 (55.6)	4 (40.0)	0.9157
Dyslipidemia	76 (91.6)	33 (91.7)	25 (89.3)	8 (88.9)	10 (100)	0.7545
Atherosclerosis obliterans	8 (9.6)	2 (5.6)	3 (10.7)	0	3 (30.0)	0.0918
Ischemic heart disease	38 (45.8)	13 (36.1)	13 (46.4)	5 (55.6)	7 (70.0)	0.2540
Atrial fibrillation	4 (4.8)	1 (2.8)	3 (10.7)	0	0	0.3326
Smoking history	63 (75.9)	28 (77.8)	18 (64.3)	8 (88.9)	9 (90.0)	0.2559
Current smoking	10 (12.1)	4 (11.1)	4 (14.3)	1 (11.1)	1 (10.0)	0.9761
Alcohol > 40 g/day	5 (6.0)	0	4 (14.3)	0	1 (10.0)	0.0881
Use of antiplatelet	81 (97.6)	35 (97.2)	27 (96.4)	9 (100)	10 (100)	0.8848
Use of anticoagulant	6 (7.2)	2 (5.6)	4 (14.3)	0	0	0.2945
Dementia	3 (3.6)	0	2 (7.1)	0	1 (10.0)	0.2772
Intracranial atherosclerosis	18 (21.7)	6 (16.7)	4 (14.3)	4 (44.4)	4 (40.0)	0.1042
**CA stenosis at examination (%)**						
Right	37.4 ± 26.3	35.5 ± 25.2	34.9 ± 30.2	51.7 ± 10.3	38.4 ± 27.7	0.4247
Left	37.4 ± 27.3	36.5 ± 29.2	45.7 ± 22.6	21.3 ± 25.9	32.2 ± 29.1	0.1811
mRS at examination	0.5 ± 0.9	0.4 ± 0.8	0.4 ± 0.8	0.4 ± 1.0	1.0 ± 1.2	0.2169
Barthel index at examination	98.0 ± 8.2	98.2 ± 5.6	99.6 ± 1.9	93.3 ± 20.0	96.5 ± 9.4	0.4729
**eGFR**						
5 y prior to examination	58.9 ± 17.1	61.7 ± 16.0	61.6 ± 17.6	56.5 ± 20.2	42.9 ± 6.5	0.0062
3 y prior to examination	58.5 ± 17.5	62.7 ± 18.8	56.9 ± 15.9	60.2 ± 18.5	45.4 ± 9.2	0.0458
At examination	58.3 ± 17.3	60.8 ± 16.5	59.7 ± 18.6	57.3 ± 19.8	46.1 ± 8.9	0.0438
**Fazekas Deep WMH grade**						
5 y prior to examination	1.0 ± 1.0	0.8 ± 0.9	1.0 ± 0.8	1.2 ± 1.1	1.8 ± 1.3	0.0391
3 y prior to examination	1.1 ± 0.9	0.8 ± 0.8	1.0 ± 0.8	1.2 ± 1.1	1.8 ± 1.2	0.0413
At examination	1.2 ± 1.0	0.9 ± 0.9	1.1 ± 0.9	1.3 ± 1.2	2.3 ± 1.1	0.0039
**Fazekas PVH grade**						
5 y prior to examination	0.8 ± 0.9	0.8 ± 0.9	0.6 ± 0.7	1.2 ± 1.2	1.1 ± 1.5	0.0978
3 y prior to examination	1.0 ± 1.0	0.9 ± 0.9	0.8 ± 0.9	1.3 ± 1.2	1.2 ± 1.4	0.2272
At examination	1.1 ± 1.0	1.0 ± 0.9	0.9 ± 0.9	1.3 ± 1.2	1.4 ± 1.5	0.1359

CA, carotid artery; mRS, modified Rankin scale; eGFR, estimated glomerular filtration rate; DWMH, deep white matter hyperintensity; PVH, periventricular hyperintensity.

### Association between eGFR or deep WMH and cognitive impairment

Table 2 shows the association between eGFR or deep WMH grade and the number of severely impaired cognitive domains. Univariate analyses revealed that eGFR and deep WMH grade associated with severe impairment in ≥3 domains or ≥2 domains, but not with severe impairment in ≥1 domain. These associations were significant for severe impairment in ≥3 domains compared to in ≥2 domains. The association was significant for eGFR at 5 years prior to examination compared to eGFR at 3 years prior to or at examination. Conversely, this association was significant for deep WMH grade at examination compared to 3 or 5 years prior to examination. No associations between the number of severely impaired cognitive domains and eGFR or deep WMH grade were seen in the reference group (data not shown).

**Table 2:** Association between eGFR or deep white matter hyperintensity and the number of severely impaired cognitive domains on Cognistat.

	Number of severely impaired domains on Cognistat								
	≥1			≥2			≥3		
	OR	95%CI	P	OR	95%CI	P	OR	95%CI	P
**eGFR (per 1-mL/min/1.73 m**^2^ **increase)**									
5 y prior to examination	0.98	0.96–1.01	0.2140	0.95	0.91–0.99	0.0045	0.89	0.82–0.97	0.0004
3 y prior to examination	0.98	0.95–1.00	0.0540	0.97	0.94–1.01	0.1020	0.94	0.88–0.99	0.0084
At examination	0.99	0.96–1.01	0.2431	0.97	0.94–1.00	0.0419	0.95	0.90–0.99	0.0116
**Fazekas deep WMH grade (per 1-grade increase)**									
5 y prior to examination	1.57	0.94–2.61	0.0755	1.89	1.09–3.29	0.0211	2.32	1.14–4.71	0.0172
3 y prior to examination	1.59	0.96–2.64	0.0611	1.99	1.15–3.47	0.0124	2.47	1.23–4.94	0.0090
At examination	1.71	1.06–2.75	0.0198	2.24	1.33–3.78	0.0015	3.32	1.61–6.85	0.0003

eGFR, estimated glomerular filtration rate; DWMH, deep white matter hyperintensity; PVH, periventricular hyperintensity; OR, odds ratio; CI, confidence interval; WMH, white matter hyperintensity.

Receiver operating characteristic analysis revealed that a cutoff eGFR of 46.8 mL/min/1.73 m^2^ at 5 years prior to examination offered the most reliable predictor of severe impairment in ≥3 domains (sensitivity 88.9%, specificity 76.5%, area under the curve (AUC) 0.848), while a cutoff of 50.4 mL/min/1.73 m^2^ at 5 years prior to examination was the most reliable predictor of severe impairment in ≥2 domains (sensitivity 70.6%, specificity 73.3%, AUC 0.741).

### Association between Cognistat scores and eGFR or deep WMH grade

**Figure 2:**
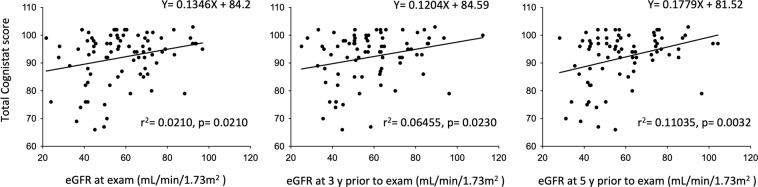
Linear association between absolute value of eGFR and total Cognistat score. The eGFR at 5 years prior to examination shows a stronger association with total Cognistat score than eGFR either at 3 years prior to examination or at examination.

Figure 2 shows linear associations between the absolute value of eGFR and total Cognistat score. The eGFR at 5 years prior to examination was significantly associated with total Cognistat score (r^2^ = 0.11035, p = 0.0032) compared to eGFR at 3 years prior to examination (r^2^ = 0.06455, p = 0.0230) or eGFR at examination (r^2^ = 0.0210, p = 0.0210). No association between eGFR and total Cognistat score was seen in the reference group (data not shown). Table 3 shows the correlation between scores for sub-components on Cognistat and eGFR. Orientation, comprehension, repetition, construction, memory, and similarity correlated significantly with eGFR at 5 years prior to examination compared to eGFR at 3 years prior to examination or eGFR at examination.

**Table 3:** Spearman’s correlation coefficient (r_s_) for eGFR and sub-component scores of Cognistat.

	eGFR at exam	eGFR 3 y prior to exam	eGFR 5 y prior to exam
Orientation	0.0964	0.1024	0.2640*
Attention	0.1291	0.1259	−0.0170
Comprehension	0.3108**	0.2595*	0.3644**
Repetition	0.2972**	0.2202*	0.3677**
Naming	−0.0081	0.0187	−0.0544
Construction	0.1948	0.2460*	0.2242*
Memory	0.0656	0.1464	0.3087**
Calculation	0.1035	0.0024	0.2158
Similarity	0.1030	0.1813	0.2789*
Judgement	0.0803	0.1676	0.0866

eGFR, estimated glomerular filtration rate

*p < 0.05; **p < 0.01.

**Figure 3:**
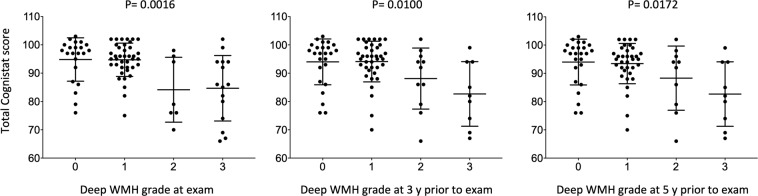
Difference in total Cognistat score between deep white matter hyperintensity (WMH) grade. Total Cognistat scores differ significantly between deep WMH grades at examination compared to deep WMH grades at 3 years or 5 years prior to examination

Figure 3 shows the difference in total Cognistat scores between deep WMH grades. Kruskal-Wallis testing revealed that total Cognistat score differed significantly between deep WMH grades at examination compared to deep WMH grades at 3 or 5 years prior to examination. On the other hand, total Cognistat score did not differ significantly between PVH grades (p = 0.816 for PVH grade at examination, p = 0.0640 for PVH grade at 3 years prior to examination, and p = 0.1570 for PVH grade at 5 years prior to examination). Table 4 shows Cognistat scores by deep WMH grade at examination. Total score, orientation, comprehension, naming, construction, calculation, similarity, and judgement were significantly lower with grade 2 or 3 compared to grade 0 or 1.

**Table 4:** Cognistat scores by deep white matter grade at examination.

	Overall	Fazekas grade for deep white matter hyperintensity				P value
		0	1	2	3	
Total score	92.0 ± 9.2	94.8 ± 7.7	94.7 ± 5.9	84.1 ± 11.5	84.7 ± 11.6	0.0016
Orientation	9.5 ± 1.2	9.9 ± 0.4	9.6 ± 1.0	9.1 ± 1.9	8.7 ± 1.8	0.0227
Attention	8.2 ± 3.0	8.6 ± 2.8	8.8 ± 2.6	6.6 ± 3.7	6.7 ± 3.6	0.0614
Comprehension	9.2 ± 1.8	9.7 ± 1.0	9.5 ± 1.4	8.3 ± 2.4	8.0 ± 2.7	0.0310
Repetition	9.7 ± 1.9	9.8 ± 2.0	9.9 ± 1.7	8.6 ± 2.3	9.5 ± 2.3	0.4406
Naming	9.6 ± 0.9	9.6 ± 0.9	9.9 ± 0.3	9.0 ± 1.0	9.3 ± 1.4	0.0085
Construction	9.0 ± 1.8	9.6 ± 1.4	9.4 ± 1.6	8.0 ± 1.7	7.4 ± 2.0	0.0009
Memory	7.8 ± 1.7	8.0 ± 1.6	7.9 ± 1.7	8.1 ± 1.8	7.2 ± 1.7	0.4436
Calculation	9.4 ± 1.4	9.6 ± 1.3	9.7 ± 1.0	8.6 ± 1.9	8.8 ± 2.0	0.0383
Similarity	9.6 ± 1.1	9.7 ± 1.0	9.8 ± 1.0	8.4 ± 1.5	9.3 ± 1.0	0.0218
Judgement	10.2 ± 1.0	10.2 ± 1.0	10.4 ± 0.9	9.4 ± 1.0	9.8 ± 1.1	0.0477

We also tested the association between deep WMH grade and eGFR. Linear regression analysis revealed that deep WMH grade at examination was not associated with eGFR (p = 0.1873 for eGFR at 5 years prior to examination, p = 0.3674 for eGFR at 3 years prior to examination, or p = 0.2705 for eGFR at examination, respectively).

## Discussion

We found that eGFR was associated with cognitive function in patients who had undergone CEA. This association was significant for eGFR at 5 years prior to cognitive examination compared to eGFR at 3 years prior to or at examination, suggesting that the effects of low eGFR might require a certain period of time to affect cognitive function, and that eGFR might be useful to predict cognitive declines in subsequent years. We also found that deep WMH was associated with cognitive declines. This association was significant for deep WMH at examination compared to deep WMH at 5 years prior to examination, suggesting that deep WMH might influence present cognitive function.

CKD has a well-established association with cognitive impairment. Impaired kidney function in the form of eGFR < 60 mL/min/1.73 m^2^ was associated with a more rapid rate of cognitive decline after adjusting for age, sex, and education in 886 elderly individuals without dementia^13^. This association persisted after excluding participants with eGFR < 30 mL/min/1.73 m^2^, indicating that the association is not limited to severe kidney dysfunction. On the other hand, cognitive decline related to kidney dysfunction was reported to improve after renal transplantation^14^, suggesting that the underlying mechanism of cognitive decline caused by kidney dysfunction is at least partially due to reversible changes rather than irreversible white matter damages. The basis for the relationship between kidney dysfunction and cognitive decline is uncertain, but the shared environmental risk factor hypothesis and the unidirectional causal hypothesis have been proposed as possible mechanisms^9^. CKD creates a toxic vascular and metabolic milieu with substances such as homocysteine, uremic toxins, creatinine, and cystatin C that promote systemic inflammation, oxidative stress, uremia, and systemic vascular endothelial dysfunction^7^. Impaired kidney function was associated with increased risk of intraplaque hemorrhage (odds ratio 1.15 per 20-point decrease in eGFR) in patients undergoing CEA, suggesting a pathway connecting CKD and WMH in patients undergoing CEA^15^.

WMH has been shown to be strongly associated with an increased risk of cognitive impairment^16^. Community-based epidemiologic studies have revealed that WMH and silent brain infarct are associated with cognitive decline^17–20^. Another study revealed WMH as one of the differences between cognitive responders and non-responders at 1 year after CEA^4^. CEA prevented the expected increase in WMH at 1 year after CEA in 14 patients with preoperative cognitive decline^1^, but long-term effects of the persistent WMH after CEA on cognition remain unknown. New ischemic lesions after carotid stenting have been shown to be associated with an increased risk of recurrent cerebrovascular events, but not after CEA^21^. WMH reduces white matter integrity, resulting in decreased coordinating interactions between different brain regions^7^. Demyelination, loss of oligodendrocytes, and axonal damage are evident in WMH, suggesting the association of chronic hypoperfusion, venous collagenosis, and damage to the blood-brain barrier^22^. These pathological changes might be associated with toxins created by CKD. The Northern Manhattan Study demonstrated that an eGFR of 15–60 mL/min/1.73 m^2^ was associated with increased log-WMH volume after adjusting for age, sex, race, and education in 615 subjects^23^. This association was not evident for eGFR > 60 mL/min/1.73 m^2^, indicating that eGFR was associated with WMH in moderate to severe CKD, but not in mild CKD. This might provide an explanation as to why WMH was not associated with eGFR in our study, where overall mean eGFR was 58 mL/min/1.73 m^2^.

Several studies have reported early changes in cognitive domains mainly at 1–6 months after CEA. Attention, memory, executive function, visuospatial orientation, psychomotor speed, and fluency have been reported to improve shortly after CEA^1,2,24–27^, while language, working memory, and global cognition deteriorated^2,4,28–30^. Only one study investigated the P300 evoked potential as a marker of cognitive function at 5 years after CEA in 25 patients, but detailed results for specific cognitive functions were not reported^31^. We showed that specific cognitive domains such as orientation, comprehension, repetition, construction, memory, and similarity correlated significantly with eGFR at 5 years prior to examination, suggesting that eGFR might also be useful to predict functional declines in these cognitive domains after several years.

A key strength of this study was that cognitive functions were assessed long after CEA, and that eGFR was measured and MRI was performed at both 5 years and 3 years before examination, but some limitations also need to be considered when interpreting the results. First, this was a retrospective cross-sectional study, not a prospective longitudinal study. We did not perform Cognistat at 5 or 3 years before examination, because Cognistat was only introduced at our institution in 2016. This makes direct evaluation of the impact of eGFR on cognitive changes difficult, but short-term longitudinal studies might be influenced by practice effects. To clarify the impact of eGFR on cognitive changes after CEA, a longitudinal study with a sufficient interval is necessary. Second, this study did not include a control group, only a reference group. Associations between eGFR or deep WMH and cognitive functions were not seen in the reference group, but this does not directly mean that these findings are specific to patients who have undergone CEA. The present findings might be more generalizable observations. Nevertheless, eGFR may offer a useful marker to predict future cognitive decline in patients who have undergone CEA.

## Conclusions

While eGFR correlated with future cognitive decline, deep WMH was associated with present cognitive function in patients who had undergone CEA. Further study is necessary to elucidate whether this association is specific after CEA or represents a generalizable phenomenon.

## Data Availability

The datasets generated and/or analyzed during the current study are available from the corresponding author on reasonable request.
